# Effectiveness of acupuncture and moxibustion combined with rehabilitation training for post-stroke shoulder-hand syndrome: a systematic review and meta-analysis

**DOI:** 10.3389/fneur.2025.1576595

**Published:** 2025-07-28

**Authors:** Huawei Gao, Zhihong Li, Wei Chen, Fangfang Shen, Yan Lu

**Affiliations:** ^1^Department of Acupuncture Moxibustion Massage Rehabilitation and Healthcare, Shandong College of Traditional Chinese Medicine, Yantai, Shandong, China; ^2^Department of Neurology, Shijingshan Teaching Hospital of Capital Medical University, Beijing Shijingshan Hospital, Beijing, China; ^3^College of Acupuncture Moxibustion and Massage, Shandong University of Traditional Chinese Medicine, Jinan, Shandong, China

**Keywords:** acupuncture, moxibustion, rehabilitation, stroke, shoulder-hand syndrome, meta-analysis

## Abstract

**Background:**

Post-stroke shoulder-hand syndrome (SHS) significantly impacts patients' quality of life and functional recovery. While both acupuncture and rehabilitation training have shown promise individually, their combined effect needs systematic evaluation.

**Methods:**

A comprehensive search was conducted across seven databases (PubMed, Embase, Cochrane Library, Web of Science, Sinomed, CNKI, and Wanfang) for randomized controlled trials comparing combined acupuncture-moxibustion-rehabilitation therapy vs. rehabilitation alone. The primary outcomes included Fugl-Meyer Assessment (FMA) scale, visual analog scale (VAS), and Barthel Index (BI) scores. Risk of bias was assessed using the Cochrane tool.

**Results:**

Twenty-seven randomized controlled trials involving 2,175 participants were included. Meta-analysis showed significant improvements in the combination therapy group compared to rehabilitation alone: VAS score (SMD = 1.62, 95% CI: 1.19–2.06), FMA scale (SMD = 1.78, 95% CI: 1.41–2.15), and BI/MBI scores (SMD = 1.01, 95% CI: 0.48–1.54). The combination therapy also showed superior effects on swelling reduction (SMD = −1.75, 95% CI: −2.08, −1.42) and total response rate (RR = 1.21, 95% CI: 1.01–1.44). Most studies demonstrated low to moderate risk of bias.

**Conclusion:**

The combination of acupuncture and moxibustion with rehabilitation training appears to be more effective than rehabilitation alone for post-stroke SHS, improving motor function, pain relief, and activities of daily living. However, high heterogeneity warrants careful interpretation and further high-quality studies.

## Introduction

Post-stroke shoulder-hand syndrome (SHS), also known as complex regional pain syndrome type 1, represents a significant challenge in stroke rehabilitation, affecting ~12%−49% of stroke survivors (1). Recent multicenter prospective studies have provided more precise regional estimates: the incidence rate was reported as 18%−42% in Nordic populations, 27% in North American cohorts, and 35% in East Asian populations (2–4). This debilitating condition typically manifests through a complex constellation of symptoms, including shoulder pain, limited range of motion, hand edema, vasomotor changes, and trophic disturbances in the affected upper limb (5).

The pathophysiology of post-stroke SHS involves intricate interactions between multiple physiological systems. The central-peripheral interaction mechanisms can be categorized into four key processes: (1) central nervous system activation leads to altered cortical reorganization and maladaptive neuroplasticity; (2) peripheral inflammatory responses trigger the release of pro-inflammatory cytokines and substance P; (3) sympathetic nervous system dysfunction results in vasomotor instability and trophic changes; (4) Autonomic nervous imbalance perpetuates the pain-inflammation-disuse cycle (6, 7). Recent neuroplasticity research indicates that acupuncture can modulate these pathways by upregulating brain-derived neurotrophic factor (BDNF) and nerve growth factor, promoting axonal regeneration and synaptic stability, while inhibiting microglial inflammatory phenotypes (8).

Traditional rehabilitation approaches have long been considered the cornerstone of SHS management. These typically include range of motion exercises, strengthening programs, neurodevelopmental techniques, and occupational therapy (9). However, conventional rehabilitation methods often show limited efficacy, particularly in managing pain and improving functional outcomes. Studies indicate that ~30%−40% of patients experience persistent symptoms despite standard rehabilitation protocols, highlighting the need for more effective therapeutic strategies (10).

In recent years, there has been growing interest in integrative approaches that combine traditional Chinese medicine (TCM) with modern rehabilitation techniques. Acupuncture and moxibustion, fundamental components of TCM, have demonstrated promising results in managing various post-stroke complications (11). These ancient therapeutic methods are believed to work through multiple mechanisms. Modern research has shown that acupuncture can modulate neural plasticity by influencing brain-derived neurotrophic factor (BDNF) levels and neuronal excitability. Additionally, both acupuncture and moxibustion have been found to regulate inflammatory mediators, enhance local blood flow, and influence pain perception pathways (12).

The theoretical basis for combining acupuncture-moxibustion with rehabilitation training lies in their complementary mechanisms of action. While rehabilitation training focuses on motor relearning and functional recovery through structured physical activities, acupuncture and moxibustion may enhance these effects by optimizing the neurophysiological environment for recovery. This combination potentially creates a synergistic effect, where the benefits of each approach are amplified when used together (13).

Despite the increasing clinical application of this combined approach and numerous individual studies reporting positive outcomes, there remains a critical need for systematic evaluation of its effectiveness. Previous systematic reviews have largely focused on either acupuncture alone or conventional rehabilitation strategies, leaving a significant gap in our understanding of the combined intervention's efficacy. Furthermore, the varying quality of available studies and potential publication bias necessitate a careful, comprehensive analysis of the existing evidence.

This systematic review and meta-analysis aims to address this knowledge gap by comprehensively evaluating the effectiveness of combining acupuncture-moxibustion with rehabilitation training compared to rehabilitation training alone for post-stroke SHS. By synthesizing the available evidence, this study seeks to provide clinicians and researchers with valuable insights into the potential benefits and limitations of this integrated approach, ultimately contributing to more effective treatment strategies for post-stroke SHS patients.

## Methods

### Protocol and registration

This systematic review was registered on INPLASY platform (INPLASY202550085) on May 15, 2025, and conducted following PRISMA 2020 guidelines.

### Search strategy and selection criteria

A comprehensive literature search was conducted across seven electronic databases: PubMed (1966-December 2024), Embase (1974-December 2024), Cochrane Library (1992-December 2024), Web of Science (1900-December 2024), Sinomed (1978-December 2024), China National Knowledge Infrastructure (CNKI, 1915-December 2024), and Wanfang Database (1990-December 2024). The search strategy combined terms related to “acupuncture,” “moxibustion,” “rehabilitation training,” and “post-stroke shoulder-hand syndrome.” All databases were searched from their inception until December 2024. The initial search yielded 135 potentially relevant articles, with 10 from PubMed, 22 from Embase, 12 from Cochrane Library, 12 from Web of Science, four from Sinomed, 41 from CNKI, and 34 from Wanfang database.

### Inclusion and exclusion criteria

The inclusion criteria were carefully defined to ensure the selection of appropriate studies. Studies were considered eligible if they were randomized controlled trials involving adult patients diagnosed with post-stroke shoulder-hand syndrome, regardless of stroke stage. The intervention of interest was acupuncture and/or moxibustion combined with rehabilitation training, compared with rehabilitation training alone as the control intervention. Eligible studies needed to report at least one of the following outcome measures: Fugl-Meyer Assessment (FMA) scale for upper extremity function, Visual analog scale (VAS) for pain assessment, Barthel Index (BI), or Modified Barthel Index (MBI) for activities of daily living, swelling scores, or total response rate (RR).

Studies were excluded from the analysis if they did not meet the randomized controlled trial design criterion, included interventions combining pharmacological agents, physical modalities, or surgical interventions beyond acupuncture/moxibustion and rehabilitation, contained duplicate publications or incomplete data, or were case reports, reviews, or animal studies. This rigorous selection process ensured the inclusion of only relevant, high-quality clinical evidence.

### Study selection and data extraction

The study selection process followed a systematic approach with two independent reviewers screening titles and abstracts according to the predefined criteria. After removing 34 duplicate articles, 101 articles remained for initial screening. The title screening process led to the exclusion of 64 articles, followed by abstract screening which eliminated an additional five articles. The remaining 32 articles underwent full-text assessment, resulting in the exclusion of five more articles. Three illustrative exclusion examples are provided in Supplementary Table S2, including both title and abstract screening examples. Five articles could not be retrieved due to journal cessation and invalid author contact information. This systematic screening process yielded a final selection of 27 studies for inclusion in the meta-analysis.

Data extraction was conducted independently by two reviewers using a standardized form designed to capture comprehensive study information. The extracted data encompassed study characteristics including author, year, and country; patient demographics such as sample size and stroke stage; detailed intervention information covering types of acupuncture/moxibustion and rehabilitation protocols; outcome measures; and follow-up duration. An example of the data extraction form is provided in Supplementary Table S1. Any disagreements encountered during the data extraction process were resolved through discussion with a third reviewer to ensure accuracy and consistency.

### Quality assessment

The methodological quality of included studies was evaluated using the Cochrane Risk of Bias tool, which assesses seven key domains of potential bias. These domains include random sequence generation, allocation concealment, blinding of participants and personnel, blinding of outcome assessment, incomplete outcome data, selective reporting, and other sources of bias. Two reviewers independently assessed each study's risk of bias, categorizing each domain as low, unclear, or high risk. Any disagreements were resolved through consensus discussion to ensure reliable quality assessment results.

### Statistical analysis

Statistical analyses were performed using Review Manager 5.4 software. For continuous outcomes including FMA, VAS, and BI/MBI scores, standardized mean differences (SMD) with 95% confidence intervals (CI) were calculated to assess treatment effects. When studies reported only median and interquartile range, mean and standard deviation were estimated using the method described by Wan et al. (14). Missing data were handled according to intention-to-treat principles. Dichotomous outcomes, such as total response rate, were analyzed using risk ratios (RR) with 95% CI. Given the expected clinical heterogeneity among studies, random-effects models were employed for all analyses.

The assessment of heterogeneity was conducted using both the *I*^2^ statistic and chi-square test. The interpretation of *I*^2^ values followed conventional thresholds, with 25%, 50%, and 75% indicating low, moderate, and high heterogeneity, respectively. When *I*^2^ exceeded 50%, sensitivity analysis was performed by excluding studies with sample sizes <40. Publication bias was assessed using Egger's test with *P* < 0.10 indicating potential bias, accompanied by funnel plot visualization. Statistical significance was set at *P* < 0.05.

Subgroup analyses were planned to explore potential sources of heterogeneity based on several key factors. These included stroke stage (I, II, or III), type of acupuncture/moxibustion technique employed, duration of treatment, and study quality. These analyses aimed to identify potential moderating factors that might influence treatment effectiveness and provide insights for clinical practice.

## Results

### Study characteristics

**Figure 1:**
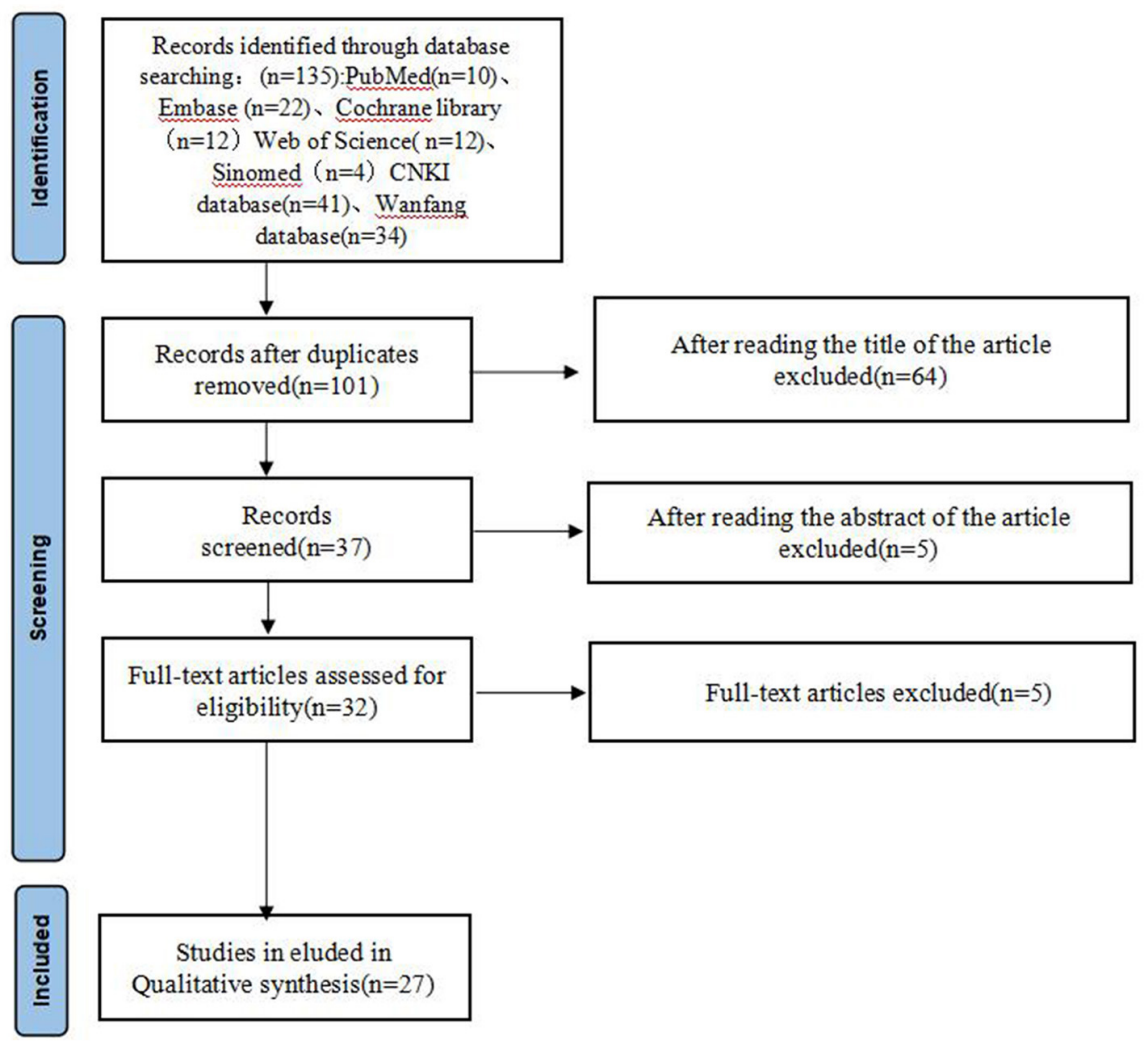
Flow diagram of literature selection process. Five articles could not be retrieved due to journal cessation and invalid author contact information.

After systematic screening, 27 randomized controlled trials meeting inclusion criteria were identified for analysis (Figure 1). These studies, published between 2004 and 2022, included a total of 2,423 participants. As shown in Tables 1a, 1b (15–41), most studies were conducted in Stage I post-stroke, with sample sizes ranging from 30 to 200 participants. The treatment interventions across studies primarily consisted of traditional manual acupuncture and moxibustion combined with rehabilitation training, though some studies utilized specific techniques such as meridian sinew row needling, White tiger head needling, and Hao-huo acupuncture.

**Table 1:** Basic study information of included literature.

**Author (year)**	**Study design**	**Sample size (E/C)**	**Stroke stage**	**Country**
Pan J (2020) (15)	RCT	30/30	Stage I, II	China
Zhan J (2022) (16)	RCT	25/24	–	China
Wang XT (2022) (17)	RCT	45/45	Stage I	China
Zou ZH (2021) (18)	RCT	58/58	Stage I	China
Gao XL (2019) (19)	RCT	24/24	–	China
Huang YS (2019) (20)	RCT	31/31	–	China
Liang H (2018) (21)	RCT	40/40	Stage III	China
Chai G (2016) (22)	RCT	59/59	Stage I	China
Chang N (2005) (23)	RCT	40/40	Stage I	China
Chen Y (2015) (24)	RCT	48/46	Stage I	China
Gao Y (2016) (25)	RCT	50/50	Stage I	China
Li S (2012) (26)	RCT	30/30	Stage I	China
Li Z (2015) (27)	RCT	46/46	Stage I	China
Liang L (2016) (28)	RCT	15/15	Stage I	China
Liao H (2006) (29)	RCT	45/45	Stage I	China
Niu G (2015) (30)	RCT	54/54	Stage I	China
Shang Y (2008) (31)	RCT	40/40	Stage I	China
Shen Z (2014) (32)	RCT	30/30	Stage I	China
Sun Y (2012) (33)	RCT	30/30	Stage I	China
Tie M (2016) (34)	RCT	50/50	Stage I	China
Wan W (2013) (35)	RCT	60/60	Stage I	China
Wang X (2017) (36)	RCT	71/71	Stage I	China
Wu Z (2014) (37)	RCT	100/100	Stage I	China
Xu F (2015) (38)	RCT	40/40	Stage I	China
Zhang X (2015) (39)	RCT	46/46	Stage I	China
Zhao X (2004) (40)	RCT	30/24	Stage I	China
Zhong Q (2011) (41)	RCT	79/79	Stage I	China

**Table 2:** Intervention details and outcome measures matrix.

**Author (year)**	**Intervention type**	**Outcome measures**
Pan J (2020) (15)	Acupuncture and moxibustion + rehabilitation	FMA, VAS
Zhan J (2022) (16)	Acupuncture and moxibustion + rehabilitation	VAS, MBI
Wang XT (2022) (17)	Meridian sinew row needling + rehabilitation	FMA, BI, VAS
Zou ZH (2021) (18)	White tiger head needling + rehabilitation	FMA, VAS, PPI
Gao XL (2019) (19)	Acupuncture and moxibustion + rehabilitation	FMA
Huang YS (2019) (20)	Hao-huo acupuncture + rehabilitation	FMA, VAS
Liang H (2018) (21)	Meridian sinew row needling + rehabilitation	FMA, VAS, BI
Chai G (2016) (22)	Acupuncture and moxibustion + rehabilitation	FMA
Chang N (2005) (23)	Acupuncture and moxibustion + rehabilitation	FMA
Chen Y (2015) (24)	Acupuncture and moxibustion + rehabilitation	FMA, VAS, BI
Gao Y (2016) (25)	Acupuncture and moxibustion + rehabilitation	VAS
Li S (2012) (26)	Acupuncture and moxibustion + rehabilitation	FMA
Li Z (2015) (27)	Acupuncture and moxibustion + rehabilitation	FMA
Liang L (2016) (28)	Acupuncture and moxibustion + rehabilitation	FMA, VAS
Liao H (2006) (29)	Acupuncture and moxibustion + rehabilitation	VAS, BI
Niu G (2015) (30)	Acupuncture and moxibustion + rehabilitation	FMA, VAS, BI
Shang Y (2008) (31)	Acupuncture and moxibustion + rehabilitation	FMA, VAS
Shen Z (2014) (32)	Acupuncture and moxibustion + rehabilitation	FMA
Sun Y (2012) (33)	Acupuncture and moxibustion + rehabilitation	FMA, VAS
Tie M (2016) (34)	Acupuncture and moxibustion + rehabilitation	FMA, BI
Wan W (2013) (35)	Acupuncture and moxibustion + rehabilitation	FMA
Wang X (2017) (36)	Acupuncture and moxibustion + rehabilitation	FMA, VAS
Wu Z (2014) (37)	Acupuncture and moxibustion + rehabilitation	FMA, VAS
Xu F (2015) (38)	Acupuncture and moxibustion + rehabilitation	FMA, VAS
Zhang X (2015) (39)	Acupuncture and moxibustion + rehabilitation	FMA, VAS
Zhao X (2004) (40)	Acupuncture and moxibustion + rehabilitation	FMA
Zhong Q (2011) (41)	Acupuncture and moxibustion + rehabilitation	FMA, BI

### Quality assessment of included studies

**Figure 2:**
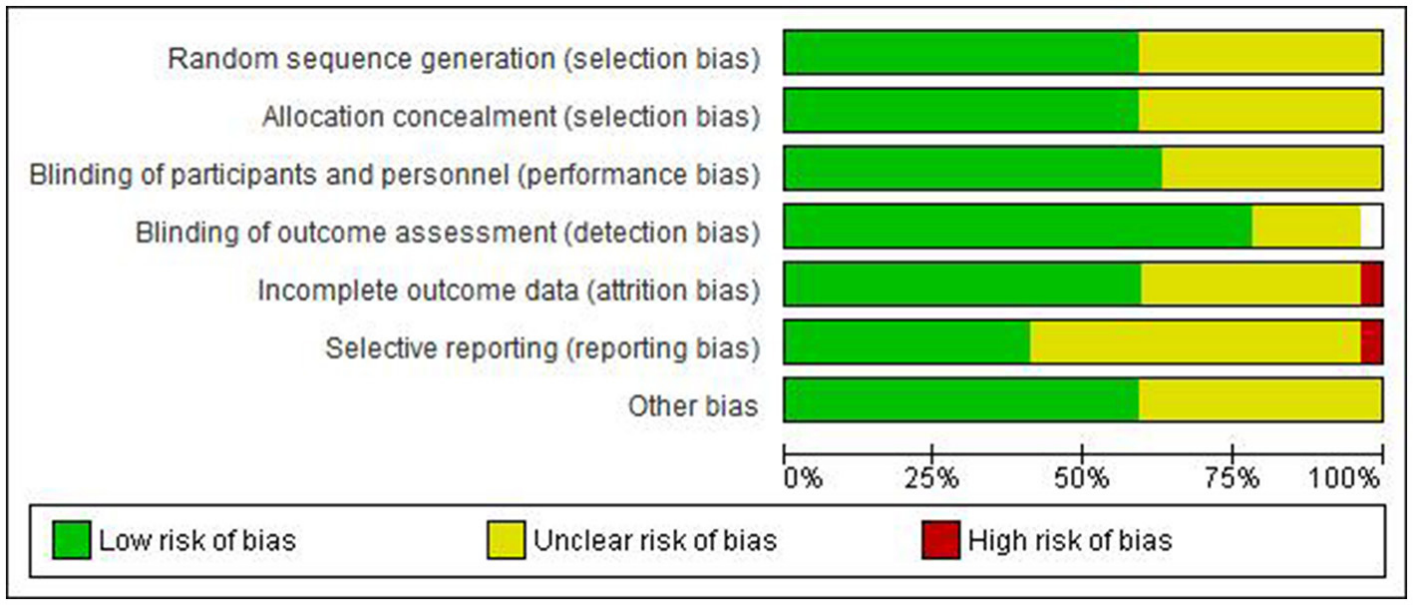
Risk of bias graph.

**Figure 3:**
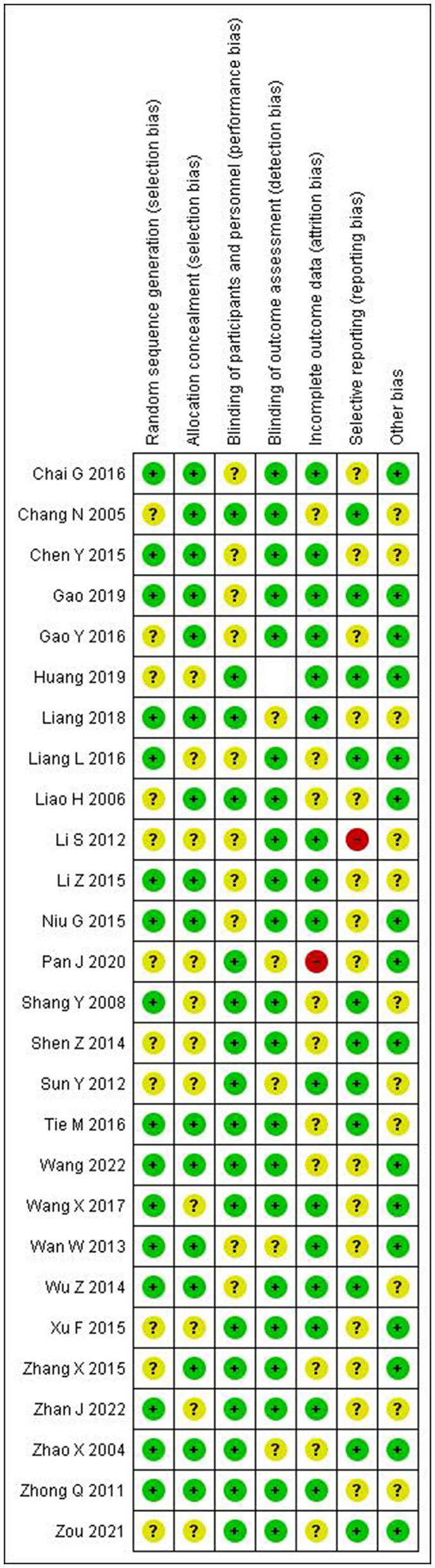
Risk of bias summary.

The quality assessment results are presented in Figures 2, 3. The majority of included studies demonstrated low to moderate risk of bias across key domains. Random sequence generation and outcome assessment were generally well-reported, showing low risk of bias. However, allocation concealment and blinding procedures were less clearly described in many studies, resulting in unclear risk assessments. Incomplete outcome data and selective reporting showed predominantly low risk of bias, indicating good quality in data handling and reporting.

### Effects on pain relief

**Figure 4:**
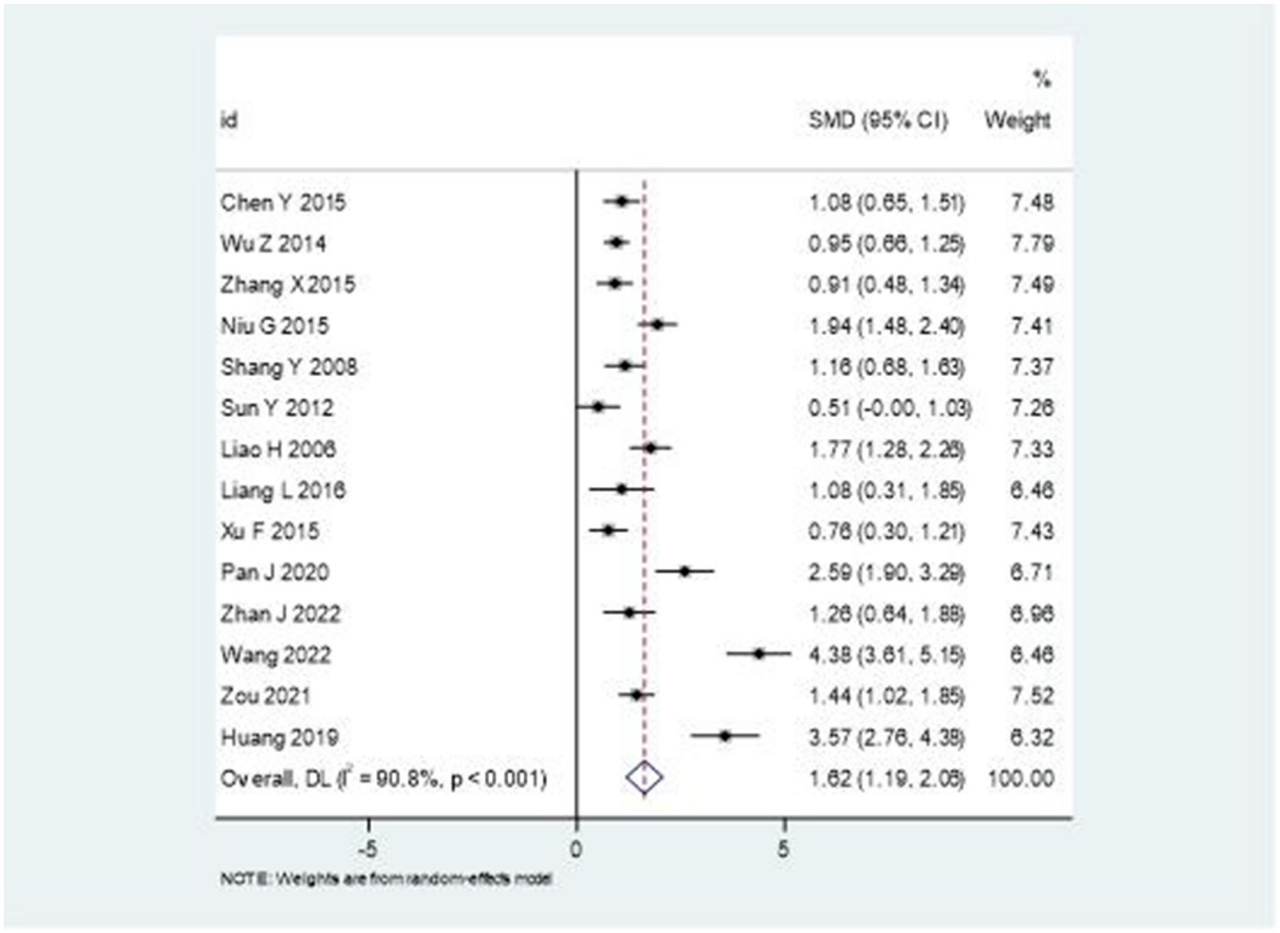
Forest plot comparing the effectiveness of combination therapy vs. rehabilitation alone on VAS scores.

Analysis of VAS scores across the studies revealed significant improvements in pain relief with combination therapy compared to rehabilitation alone. As shown in Figure 4, the pooled standardized mean difference was 1.62 (95% CI: 1.19–2.06), favoring the intervention group. However, significant heterogeneity was observed (*I*^2^ = 90.8%, *P* < 0.001), suggesting considerable variation in treatment effects across studies. After excluding studies with sample sizes <40, the *I*^2^ value decreased to 55%, with the effect size remaining stable.

### Upper extremity motor function

**Figure 5:**
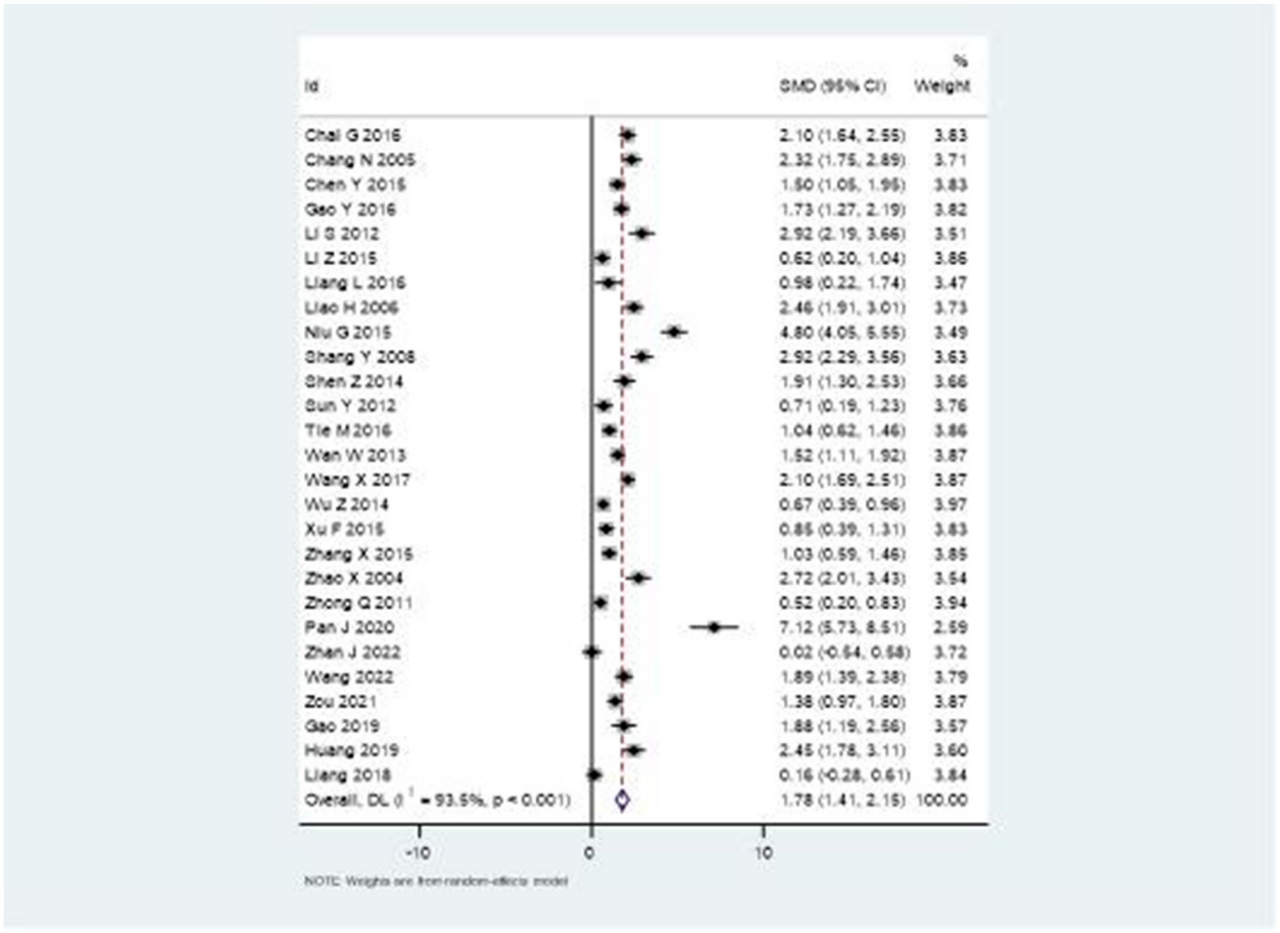
Forest plot of Fugl-Meyer Assessment scale score changes.

The Fugl-Meyer Assessment (FMA) scale results demonstrated substantial improvement in upper extremity function with combination therapy. Figure 5 illustrates the forest plot analysis, showing a significant pooled SMD of 1.78 (95% CI: 1.41–2.15). This robust effect size indicates meaningful clinical improvement, though high heterogeneity was noted (*I*^2^ = 93.5%, *P* < 0.001).

### Activities of daily living

**Figure 6:**
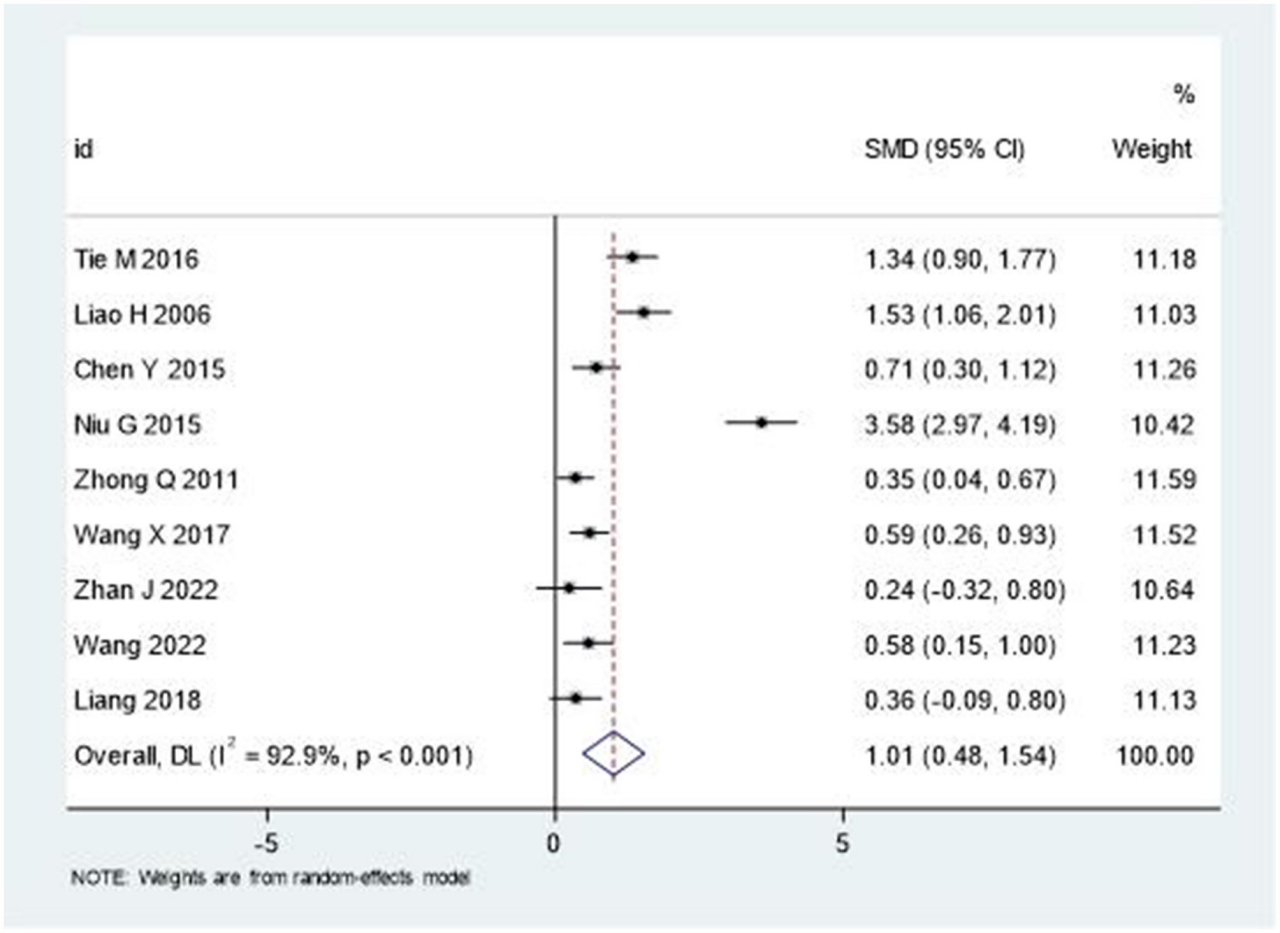
Forest plot of Barthel Index changes.

The analysis of Barthel Index (BI) and Modified Barthel Index (MBI) scores demonstrated significant improvements in activities of daily living. As depicted in Figure 6, the pooled SMD was 1.01 (95% CI: 0.48–1.54), indicating moderate to large treatment effects. Similar to other outcomes, substantial heterogeneity was observed (*I*^2^ = 92.9%, *P* < 0.001).

### Swelling reduction

**Figure 7:**
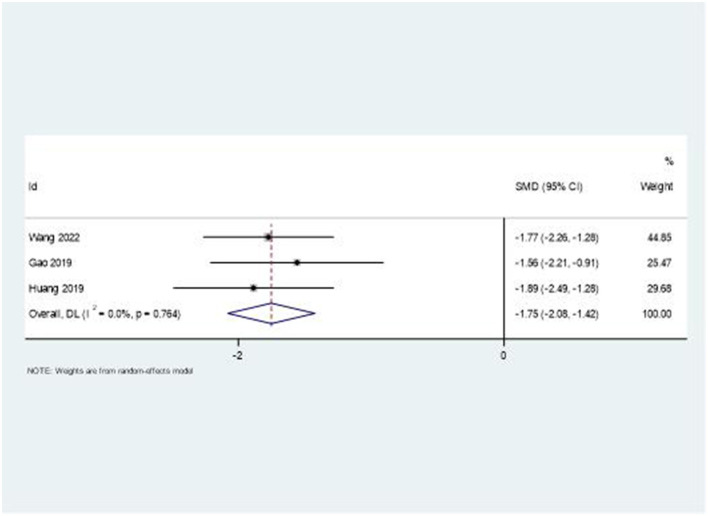
Forest plot of swelling score changes.

The combination therapy showed remarkable effectiveness in reducing upper limb swelling. Figure 7 presents the forest plot analysis, revealing a large effect size with SMD = −1.75 (95% CI: −2.08, −1.42). Notably, this outcome demonstrated low heterogeneity (*I*^2^ = 0%, *P* = 0.764), suggesting consistent treatment effects across studies.

### Total response rate

**Figure 8:**
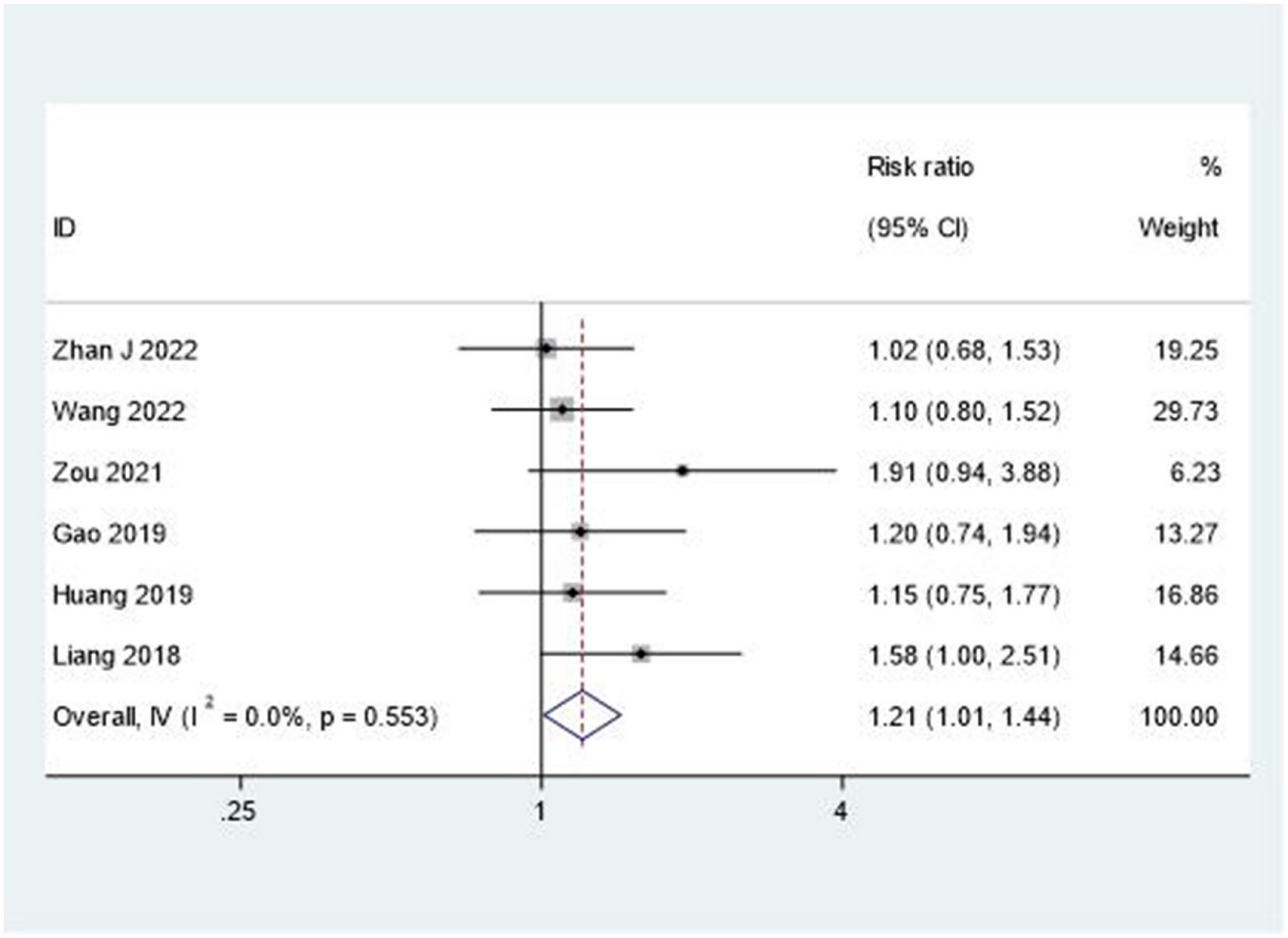
Forest plot of total response rate.

The overall therapeutic effectiveness was evaluated through total response rate analysis. As shown in Figure 8, the combination therapy demonstrated superior outcomes compared to rehabilitation alone (RR = 1.21, 95% CI: 1.01–1.44). This analysis showed minimal heterogeneity (*I*^2^ = 0%, *P* = 0.553), indicating consistent treatment benefits across studies.

## Discussion

This systematic review and meta-analysis provides comprehensive evidence supporting the effectiveness of combining acupuncture and moxibustion with rehabilitation training for post-stroke shoulder-hand syndrome. Our analysis of 27 randomized controlled trials reveals significant improvements across multiple outcome measures, warranting detailed discussion of the implications, limitations, and future directions for clinical practice and research. In the acute phase ( ≤ 1 month), treatment priorities should focus on pain relief and improving blood flow, while subacute and chronic phases should emphasize joint range of motion and hand function training. The studies included in this review primarily focused on patients in the early inpatient rehabilitation phase (average 2–8 weeks post-stroke), thus our conclusions are most applicable to the subacute rehabilitation stage and should not be directly extrapolated to community-based chronic care settings.

The substantial improvement in pain relief, as evidenced by the VAS score analysis (SMD = 1.62, 95% CI: 1.19–2.06), represents a clinically meaningful outcome. This finding aligns with recent systematic reviews demonstrating that acupuncture modulates pain perception through multiple neurophysiological mechanisms, including endogenous opioid release and modulation of inflammatory mediators (42). The combination with rehabilitation training may create a synergistic effect, where reduced pain facilitates more effective participation in rehabilitation exercises, potentially leading to better functional outcomes.

The marked improvement in upper extremity motor function, demonstrated by the FMA scale results (SMD = 1.78, 95% CI: 1.41–2.15), is particularly noteworthy. This substantial effect size suggests that the combination therapy may enhance neuroplasticity and motor recovery more effectively than rehabilitation alone. Recent neuroimaging studies have shown that acupuncture can modulate cortical reorganization and enhance motor pathway connectivity (43), which may explain the superior outcomes when combined with structured rehabilitation protocols.

The significant improvement in activities of daily living, as measured by BI/MBI scores (SMD = 1.01, 95% CI: 0.48–1.54), has important implications for patient independence and quality of life. This improvement may be attributed to the cumulative benefits of enhanced motor function and reduced pain, allowing patients to engage more effectively in daily activities. The economic implications of improved functional independence, including reduced caregiving needs and healthcare costs, warrant further investigation (44).

A particularly striking finding was the consistent effect on swelling reduction (SMD = −1.75, 95% CI: −2.08, −1.42) with remarkably low heterogeneity. This suggests that the combination therapy may effectively address the inflammatory and autonomic components of SHS. Traditional Chinese medicine theory proposes that acupuncture and moxibustion improve local circulation and reduce inflammation, a concept increasingly supported by modern research demonstrating their effects on inflammatory markers and microcirculation (45).

When compared with other treatment modalities, our combined approach shows superior pain relief efficacy (SMD 1.62) compared to NSAIDs or physical modalities alone (typical SMD 0.4–0.7), with minimal adverse effects. Future research should focus on developing standardized “acupuncture prescription + rehabilitation dosage” pathways and conducting comprehensive cost-effectiveness analyses. Recent network meta-analyses have provided supporting evidence for the superiority of combined approaches over monotherapies in stroke rehabilitation (46).

While our findings are promising, several methodological considerations deserve attention. The high heterogeneity observed in some outcome measures (*I*^2^ > 90% for VAS, FMA, and BI/MBI) suggests substantial variation in treatment effects across studies. This heterogeneity may be attributed to several factors, including differences in acupuncture techniques, treatment duration, practitioner expertise, and patient characteristics (47).

The quality assessment revealed varying levels of methodological rigor across studies. While random sequence generation was generally well-reported, inadequate description of allocation concealment and blinding procedures in many studies raises concerns about potential selection and performance bias. This limitation is common in acupuncture research, where complete blinding of practitioners is inherently challenging (48).

This review has several limitations that should be acknowledged. First, the systematic review was not prospectively registered on PROSPERO, representing a *post-hoc* registration limitation, though we adhered to PRISMA 2020 guidelines throughout the process. Second, the predominance of studies conducted in Stage I stroke patients limits the generalizability of findings to later stages of recovery. Additionally, the variation in rehabilitation protocols across studies makes it difficult to isolate the specific contributions of different components of the combination therapy (49).

Several key areas warrant further investigation. First, there is a need for large-scale, multicenter trials with rigorous methodology to validate these findings. Such studies should incorporate standardized protocols for both acupuncture and rehabilitation components, allowing for better assessment of treatment fidelity and effectiveness (50).

Long-term follow-up studies are essential to evaluate the sustainability of treatment effects and potential impact on stroke recovery trajectories. Future research should also explore the optimal timing, frequency, and duration of combination therapy, as well as potential predictors of treatment response.

The development of more objective outcome measures, including biomarkers and advanced imaging techniques, could provide deeper insights into the mechanisms underlying the observed clinical benefits. Integration of cost-effectiveness analyses would also be valuable for healthcare policy and resource allocation decisions.

Based on our findings, several recommendations can be made for clinical practice. The combination of acupuncture and moxibustion with rehabilitation training should be considered as a viable treatment option for post-stroke SHS, particularly in early stages. However, practitioners should carefully consider individual patient factors and standardize treatment protocols to the extent possible.

Regular assessment using validated outcome measures is crucial for monitoring treatment progress and adjusting interventions accordingly. The relatively low risk of adverse effects reported in the included studies suggests a favorable safety profile, though careful patient selection and monitoring remain important.

The implementation of this combination therapy requires integration of traditional Chinese medicine practitioners with rehabilitation specialists, highlighting the need for interdisciplinary collaboration and standardized training programs. Development of clinical guidelines incorporating both Eastern and Western approaches could help optimize treatment delivery and outcomes.

## Conclusion

This systematic review and meta-analysis demonstrates that the combination of acupuncture and moxibustion with rehabilitation training provides superior therapeutic benefits compared to rehabilitation alone for post-stroke shoulder-hand syndrome. The combined intervention showed significant improvements across multiple outcome measures, including pain reduction (VAS scores), motor function recovery (FMA scale), activities of daily living (BI/MBI scores), and swelling reduction. The consistently positive results across different outcome measures, particularly the marked improvement in motor function and notable reduction in swelling with low heterogeneity, suggest this combination therapy could be an effective treatment strategy.

## Data Availability

The original contributions presented in the study are included in the article/Supplementary material, further inquiries can be directed to the corresponding author.

## References

[1] Zhan J Zhang P Wen H Wang Y Yan X Zhan L . Global prevalence estimates of poststroke fatigue: a systematic review and meta-analysis. Int J Stroke. (2023) 18:1040–50. 10.1177/17474930221138701 36314998

[2] Lindgren I Jönsson AC Norrving B Lindgren A. Shoulder pain after stroke: a prospective population-based study. Stroke. (2007) 38:343–8. 10.1161/01.STR.0000254598.16739.4e 17185637

[3] Adey-Wakeling Z Arima H Crotty M Leyden J Kleinig T Anderson CS . Incidence and associations of hemiplegic shoulder pain post-stroke: prospective population-based study. Arch Phys Med Rehabil. (2015) 96:241–7.e1. 10.1016/j.apmr.2014.09.007 25264111

[4] Li Y Yang S Cui L Bao Y Gu L Pan H . Prevalence, risk factor and outcome in middle-aged and elderly population affected by hemiplegic shoulder pain: An observational study. Front Neurol. (2023) 13:1041263. 10.3389/fneur.2022.1041263 36712437 PMC9879055

[5] Cheng Y Wu B Huang J Chen Y. Research progress on the mechanisms of central post-stroke pain: a review. Cell Mol Neurobiol. (2023) 43:3083–98. 10.1007/s10571-023-01360-6 37166685 PMC11409963

[6] Pertoldi S Di Benedetto P. Shoulder-hand syndrome after stroke: a complex regional pain syndrome. Eura Medicophys. (2005) 41:283–92. 16474282

[7] Israely S Leisman G Carmeli E. Neuromuscular synergies in motor control in normal and poststroke individuals. Rev Neurosci. (2018) 29:593–612. 10.1515/revneuro-2017-0058 29397390

[8] Qin S Zhang Z Zhao Y Liu J Qiu J Gong Y . The impact of acupuncture on neuroplasticity after ischemic stroke: a literature review and perspectives. Front Cell Neurosci. (2022) 16:817732. 10.3389/fncel.2022.817732 36439200 PMC9685811

[9] van Mastrigt G van Heugten C Visser-Meily A Bremmers L Evers S. Estimating the burden of stroke: two-year societal costs and generic health-related quality of life of the Restore4Stroke Cohort. Int J Environ Res Public Health. (2022) 19:11110. 10.3390/ijerph191711110 36078828 PMC9517815

[10] Li X He Y Wang D Rezaei MJ. Stroke rehabilitation: from diagnosis to therapy. Front Neurol. (2024) 15:1402729. 10.3389/fneur.2024.1402729 39193145 PMC11347453

[11] Guo C Wang Y Wang S Zhang S Tai X. Effect and mechanism of traditional Chinese medicine exercise therapy on stroke recovery. Evid Based Complement Alternat Med. (2023) 2023:5507186. 10.1155/2023/5507186 36865742 PMC9974248

[12] Zhang J Lu C Wu X Nie D Yu H. Neuroplasticity of acupuncture for stroke: an evidence-based review of MRI. Neural Plast. (2021) 2021:2662585. 10.1155/2021/2662585 34456996 PMC8397547

[13] Noh J Lim J Shin JC Bang S-P Noh G Yoon J-S . Research trends on immune mechanisms of acupuncture: a literature review. J Acupunct Res. (2023) 40:329–43. 10.13045/jar.2023.00248

[14] Wan X Wang W Liu J Tong T. Estimating the sample mean and standard deviation from the sample size, median, range and/or interquartile range. BMC Med Res Methodol. (2014) 14:135. 10.1186/1471-2288-14-135 25524443 PMC4383202

[15] Pan J Cao Y Fang C Zhang Y Tang JZ Zhang W . Clinical efficacy observation of acupuncture combined with rehabilitation training for post-stroke shoulder-hand syndrome. World J Acupunct Moxibust. (2020) 30:107–12. 10.1016/j.wjam.2020.05.015

[16] Zhan J Ai Y Zhan L Pan R Wang Y Dong C . Effect of abdominal acupuncture combined with routine rehabilitation training on shoulder-hand syndrome after stroke: a randomized controlled trial. Integr Med Res. (2022) 11:100805. 10.1016/j.imr.2021.100805 34877254 PMC8627967

[17] Wang XT Liu HF Huang HQ Fang F. Clinical study of Yang meridian acupuncture combined with rehabilitation training for stroke-related shoulder-hand syndrome. New Trad Med. (2022) 54:195–9. 10.13457/j.cnki.jncm.2022.07.046

[18] Zou ZH Wu YL Lu J Wen J. Clinical effect of Baihu Yaotou acupuncture combined with rehabilitation on post-stroke shoulder-hand syndrome pain and upper limb function. Clin J Acupunct. (2021) 37:19–25. 10.19917/j.cnki.1005-0779.021238

[19] Gao XL. Clinical effect of different acupuncture therapies combined with rehabilitation training on post-stroke shoulder-hand syndrome. Chin-West Cardiovasc Dis Electron J. (2019) 7:172–8. 10.16282/j.cnki.cn11-9336/r.2019.23.141

[20] Huang YS Zhuo YY Yu HB. Clinical efficacy of needle fire combined with rehabilitation training for post-stroke shoulder-hand syndrome. Inner Mongolia J Tradit Chin Med. (2019) 38:104–6. 10.16040/j.cnki.cn15-1101.2019.09.063

[21] Liang H Li ZR Chen JW Lin HB. Clinical study of giant-needle acupuncture combined with rehabilitation training for post-stroke shoulder-hand syndrome in the third phase. Clin J Acupunct. (2018) 34:19–23. 10.3969/j.issn.1005-0779.2018.08.007

[22] Chai GH. Acupuncture combined with rehabilitation training for 59 patients with shoulder-hand syndrome. Chin Nat Med. (2016) 24:35–6. 10.19621/j.cnki.11-3555/r.2016.03.026

[23] Chang N Liu S. Therapeutic effect of acupuncture combined with rehabilitation exercise in patients with shoulder-hand syndrome. J Fourth Mil Med Univ. (2005) 26:2295–7. 10.3321/j.issn:1000-2790.2005.24.02725069201

[24] Chen Y Huang TS Liu KC. Clinical research of acupuncture combined with rehabilitation training in the treatment of post-stroke shoulder-hand syndrome. Sichuan J Tradit Chin Med. (2015) 33:150–2.

[25] Gao Y. Effectiveness evaluation of acupuncture combined with rehabilitation exercise for shoulder-hand syndrome. Guide Chin Med. (2016) 14:198. 10.15912/j.cnki.gocm.2016.28.163

[26] Li SZ Wang WY Liu J. Acupuncture combined with rehabilitation training for shoulder-hand syndrome after stroke. Chin J Tradit Med Sci Technol. (2012) 19:552–3. 10.3969/j.issn.1005-7072.2012.06.055

[27] Li ZF Gu JH Hu M. Clinical observation of acupuncture for 46 patients with shoulder-hand syndrome after stroke. Chin J Ethnomed Ethnophar. (2015) 24:78–9. 10.3969/j.issn.1007-8517.2015.21.047

[28] Liang LN Zhang YJ Liu YH. Acupuncture combined with rehabilitation training for 30 patients with post-stroke shoulder-hand syndrome. Lab Med. (2016) 31:203.

[29] Liao HW. Clinical observations on the efficacy of occupational therapy plus acupuncture for treating reflex sympathetic dystrophy. Shanghai J Acupunct Moxibust. (2006) 25:9–10. 10.13460/j.issn.1005-0957.2006.03.005

[30] Niu GP. Clinical study of acupuncture combined with rehabilitation training for shoulder-hand syndrome. Henan Trad Chin Med. (2015) 35:2846–7. 10.16367/j.issn.1003-5028.2015.11.1220

[31] Shang YJ Ma CC Cai YY Wang D-S Kong L-L. Clinical study on acupuncture combined with rehabilitation therapy for treatment of post-stroke shoulder-hand syndrome. Chin Acupunct Moxibust. (2008) 28:331–3. 18652322

[32] Shen ZH. Clinical observation of acupuncture combined with rehabilitation training for shoulder-hand syndrome. Shenzhen J Integr Trad Chin West Med. (2014) 24:56–8. 10.16458/j.cnki.1007-0893.2014.08.007

[33] Sun YZ Wang YJ Wang W. Effect of acupuncture plus rehabilitation training on shoulder-hand syndrome due to ischemic stroke. J Acupunct Tuina Sci. (2012) 10:109–13. 10.1007/s11726-012-0583-z

[34] Tie M. Effectiveness of rehabilitation training for shoulder-hand syndrome after stroke. Contemp Med Symp. (2016) 14:144–5.

[35] Wan WR Wang TL Cheng SL Zhao Y-L Zhang W Wu Q-Y . Post-stroke shoulder-hand syndrome treated with acupuncture and rehabilitation: a randomized controlled trial. Chin Acupunct Moxibust. (2013) 33:970–4. 10.13703/j.0255-2930.2013.11.026 24494280

[36] Wang XQ Gao Y Gao S. Acupuncture combined with rehabilitation on the influence of PRI, FMA, and MBI in patients with shoulder-hand syndrome after stroke. Glob Trad Chin Med. (2017) 10:361–3. 10.3969/j.issn.1674-1749.2017.03.033

[37] Wu ZG. Acupuncture combined with rehabilitation training for 100 patients with shoulder-hand syndrome after stroke. Chin Med Mod Dist Educ China. (2014) 12:81–2. 10.3969/j.issn.1672-2779.2014.20.046

[38] Xu F Li HL Zhang Q. Acupuncture combined with rehabilitation training for shoulder-hand syndrome after ischemic stroke: a randomized controlled trial. J Trauma Disabil Med. (2015) 23:141–2. 10.13214/j.cnki.cjotadm.2015.16.107

[39] Zhang XR. Clinical effectiveness analysis of acupuncture combined with rehabilitation training for shoulder-hand syndrome after stroke. China Med Eng. (2015) 23:200.

[40] Zhao XF Song H. Acupuncture for 30 patients with shoulder-hand syndrome after stroke. Chin J Inf Trad Med. (2004) 11:532–3.

[41] Zhong Q Feng QH Yi G. Synthetic rehabilitation therapy for shoulder-hand syndrome after stroke. Pract J Clin Med. (2011) 8:115–6. 10.3969/j.issn.1005-5304.2004.06.037

[42] Chen Y Liu Y Song Y Zhao S Li B Sun J . Therapeutic applications and potential mechanisms of acupuncture in migraine: a literature review and perspectives. Front Neurosci. (2022) 16:1022455. 10.3389/fnins.2022.1022455 36340786 PMC9630645

[43] Onose G Anghelescu A Blendea CD Ciobanu V Daia CO Firan FC . Non-invasive, non-pharmacological/bio-technological interventions towards neurorestoration after ischemic stroke: a systematic, synthetic literature review. Front Biosci. (2021) 26:1204–39. 10.52586/5020 34856764

[44] Rajsic S Gothe H Borba HH Sroczynski G Vujicic J Toell T . Economic burden of stroke: a systematic review on post-stroke care. Eur J Health Econ. (2019) 20:107–34. 10.1007/s10198-018-0984-0 29909569

[45] Zhang B Shi H Cao S Xie L Ren P Wang J . Revealing the magic of acupuncture based on biological mechanisms: a literature review. Biosci Trends. (2022) 16:73–90. 10.5582/bst.2022.01039 35153276

[46] Shi J Chen F Liu Y Bian M Sun X Rong R . Acupuncture versus rehabilitation for post-stroke shoulder-hand syndrome: a systematic review and meta-analysis of randomized controlled trials. Front Neurol. (2025) 16:1488767. 10.3389/fneur.2025.1488767 40242619 PMC12000064

[47] Ho L Ke FYT Wong CHL Wu IXY Cheung AKL Mao C . Low methodological quality of systematic reviews on acupuncture: a cross-sectional study. BMC Med Res Methodol. (2021) 21:1–11. 10.1186/s12874-021-01437-0 34717563 PMC8557536

[48] Liu T Jiang L Li S Cheng S Zhuang R Xiong Z . The blinding status and characteristics in acupuncture clinical trials: a systematic review and meta-analysis. Syst Rev. (2024) 13:302. 10.1186/s13643-024-02692-0 39643890 PMC11624600

[49] Kwakkel G Lannin NA Borschmann K English C Ali M Churilov L . Standardized measurement of sensorimotor recovery in stroke trials: consensus-based core recommendations from the Stroke Recovery and Rehabilitation Roundtable. Int J Stroke. (2017) 12:451–61. 10.1177/1747493017711813 28697709

[50] Zhu RR Wang JX Pan B Lai HH Xu XT Ge L . Evidence-based evaluation for stroke guidelines mentioning traditional and complementary medicine rehabilitation. BMC Complement Med Ther. (2025) 25:1–12. 10.1186/s12906-025-04916-9 40390019 PMC12090438

