# Supplementary material for: Effectiveness of acupuncture and moxibustion combined with rehabilitation training for post-stroke shoulder-hand syndrome: a systematic review and meta-analysis
Source: Front Neurol. 2025 Jul 28;16:1576595. doi: 10.3389/fneur.2025.1576595 (PMC12337482; doi:10.3389/fneur.2025.1576595)
Supplement: Supplementary file 1 [file Table_1.docx]

# Supplementary Table S1. Data‑Extraction Template (Example First Row)

| Study ID | First author | Year | Country/Region | Design | Sample size (n) | Mean age (y) / % male | Stroke phase* | Intervention (details) | Control | Primary outcome(s) |
| --- | --- | --- | --- | --- | --- | --- | --- | --- | --- | --- |
| R1 | Lindgren | 2007 | Sweden | Prospective cohort | 142 | 72.4 / 49.3% | Sub‑acute (2–12 wk) | Manual acupuncture 3× wk × 4 wk ‘Jian Yu’, ‘Jian Liao’, ‘LI14’ | Conventional rehab only | VAS shoulder pain; FMA‑UE |
