# Supplementary material for: Effectiveness of acupuncture and moxibustion combined with rehabilitation training for post-stroke shoulder-hand syndrome: a systematic review and meta-analysis
Source: Front Neurol. 2025 Jul 28;16:1576595. doi: 10.3389/fneur.2025.1576595 (PMC12337482; doi:10.3389/fneur.2025.1576595)
Supplement: Supplementary file 2 [file Table_2.docx]

# Supplementary Table S2. Title/Abstract & Full‑Text Screening Exclusion Examples

| Stage | Citation (First author, Year) | Reason for Exclusion | Notes |
| --- | --- | --- | --- |
| Title/Abstract | Gordon 2016 | Not stroke population (frozen shoulder) | — |
| Title/Abstract | Kim 2019 | Protocol only; no outcome data | Registered RCT |
| Full text | Pradhan 2014 | Combined acupuncture with NSAIDs (co‑intervention) | Failed pre‑defined criteria |
